# Efficient Denoising Framework for Mammogram Images with a New Impulse Detector and Non-Local Means

**DOI:** 10.31557/APJCP.2020.21.1.179

**Published:** 2020

**Authors:** Harikumar Rajaguru, Sannasi Chakravarthy S R

**Affiliations:** *Department of Electronics and Communication Engineering, Bannari Amman Institute of Technology, India. *

**Keywords:** Mammogram, impulse noise, denoising framework, non-local means filter, adaptive fuzzy median filter

## Abstract

**Objective:**

The survival rates of breast cancer are increasing as screening and diagnosis improve. The removal of noise is revealed to be a significant step for automatic - computer aided detection (CAD) of microcalcification in digital mammography.

**Methods:**

In this paper, a combined approach for eradicating impulse noise from digital mammograms is proposed. The process is achieved in two stages, detection of noise followed by filtering of noise. The detection of noise is carried out by using Modified Robust Outlyingness Ratio (mROR) trailed by an extended NL (Non-Local)-means filter for filtering mechanism.

**Results:**

According to the value of mROR, all pixels in mammogram images are divided into four distinct groups. In each cluster, many decision rules are then applied for detecting the impulse noise. Filtering is done with NL-means filter by providing a reference mammogram image.

**Conclusion:**

The comparative analysis and evaluated results are compared with some existing filters which indicate that the proposed structure outperforms the analysed result of others.

## Introduction

Breast cancer is the most invasive cancer among today’s women and its associated risk increases with age (Siegel at al., 2017). Generally it occurs in women and very rare for men. It leads to the cause of cancer death prominently over the age of 40 among women. Since the exact cause of breast cancer remains unclear, deterrence of this type of cancer is difficult (DeSantis et al., 2017). But its earlier detection and removal of disease can increase the survival rate. The survival rate of breast cancer is higher in developed countries than in the developing countries (Siegel at al., 2015). The earlier detection of tumours in breast can be done by Mammography which is the most effective and reliable screening tool (Sannasi Chakravarthy et al., 2019). Mammography refers to the process of creating breast images by means of exposing low-energy x-rays of around 30 kVp to study the breast tumour for screening and diagnosis (Gøtzsche and Nielsen, 2009). It aims to detect the breast cancer at earlier usually through the revealing of microcalcification. It is used to check for any lump or other sign of breast cancer and also used for the screening of breast cancer in women with no signs or symptoms of tumour (Subhasakthe et al., 2015). 

During image acquisition or transmission, impulse noise can distorts the obtained mammogram images. The impulse noise can affect the images by padding random additional values to some pixels in the obtained raw image (Wang and Zhang, 1999). Many preprocessing stages are involved to increase the mammogram image quality and so it will be prepared for further processing of diagnosis. Hence the noise removal plays a vital role in preprocessing stage during the breast cancer diagnosis in CAD system.

The removal of impulse noise can be done by using any non-linear filters since the impulse noise distorts only a few region of pixels in the obtained mammogram image. The median based and modified median based non-linear filters like weighted median filter (Yin et al., 1996), center weighted median filter (Chan et al., 2005), multistate median filter (Zhang et al., 2014) were introduced for the better performance of noise removal. The primary drawback of these filters is that they can perform the role of filtering throughout the image deprived of inspecting whether the present pixel is distorted or not. Thus at the end, these filters confiscate the desired details of the entire mammogram image. This affects the further processing of mammogram images and leads to the degradation of image quality particularly for the image with higher noise density. The switching median filters (Zhang and Wang, 2015) were developed in order to overcome this limitation. To determine the corrupted pixels and to leave the uncorrupted pixels, this method utilizes a noise detection technique with median filtering framework.

This paper proposed an mROR (Modified Robust Outlyingness Ratio) based statistical detector for computing the noise level in each pixel of mammogram image. All the pixels in the obtained mammogram image are grouped into four distinct clusters based on the value of mROR and various decision algorithms are then incorporated in each cluster to identify the impulse noise in the image. Now after identifying the noisy pixels, the NL-means filter is extended to rectify the noisy pixels. Thus this method removes the noise in the corrupted pixels effectively without disturbing the uncorrupted pixels.

**Figure 1:**
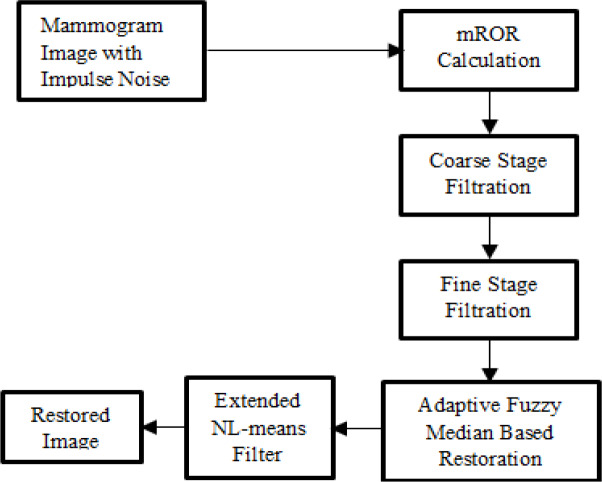
Proposed Work for Noise Removal

Figure 1 shows the stages involved to remove noise and to restore original image in proposed method. Calculation of value of mROR is done first and divide the pixels into four distinct clusters based on the value of obtained mROR. And in each cluster, impulse noise is detected separately. Different decision rules are then applied with different thresholds in each cluster. For filtering, coarse and fine stage are carried out. Finally extended NL-means filter is used for the mammogram image restoration. 

## Materials and Methods

*A. Impulse Noise Model*

Impulse noise is often occurred during the acquisition, transmission, storage and processing of obtained mammogram images. The occurrence of impulse noise may be either comparatively high or low in an image. Hence it can worsen the image quality rigorously and it will leads to loss of image information details for diagnosis. Moreover it doesn’t affects all the pixels in an image; it only distorts some of the pixels in any region of the image (Xiong and Yin, 2012). Due to impulse noise, some pixels in an image are arbitrarily misfired and distorted with other values in an image (Coles et al., 1968). The impulse noise model in an image is described as:


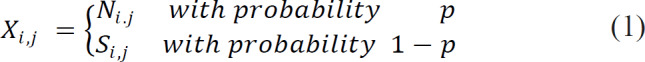


where *S*_i,j_ indicates the pixel in noiseless original image and *N*_i,j_ represents the noisy pixel altered in place of original pixel in an image. The rate at which the image is distorted by impulse noise is given by the parameter* p*. 

The most common impulse noise models of mammogram images are fixed-valued and random-valued impulse noise models. The fixed-valued impulse noise model (salt-and pepper noise) is comprised of corrupted pixels in which the values are altered with either maximum (*η*_max_) or minimum (*η*_min_) of the permissible pixel range whereas the random-valued impulse noise model is made of corrupted pixels in which its values are replaced uniformly between the maximum (*η*_max_) and minimum (*η*_min_) of the allowable pixel range. The removal of random-valued impulse noise is quite more tedious than the removal of fixed-valued impulse noise. This is due to computing difference value of the pixels between a noise affected pixel and its uncorrupted neighbor pixels are frequently important in cleaning the random-valued impulse noise (Nikolova, 2004).

*B. Modified Robust Outlyingness Ratio (mROR)*

The mechanism of removing impulse noise is based on two state methods which indicates each pixel in an image as either corrupted or uncorrupted ones (Garnett et al., 2005). Its primary goal is to find pixels that are significant outliers while comparing with their adjacent pixels in an image. This offers the advantage of integration of noise detection technique with filtering mechanism. This allows only the pixels detected as noisy to the filtering process and the identified noise-free pixels remain undisturbed. 

This can be achieved simply through comparing intensity level of the pixel with its neighbor pixel’s median intensity (Aizenberg and Butakoff, 2004). Also many advanced modifications have been proposed. But these methods has a primary limitation that each pixel is determined based on the similar decision rule without considering how much impulse-like each pixel is (Marghny and Taloba, 2014). Moreover the performance of these methods are poor for higher noise density (Sreedevi and Sherly, 2015). 

This paper proposed a noise detection mechanism to identify the level of noise in each pixel based on a statistics mROR. The traditional statistical method measures the outlyingness with respect to a sample which is based on the sample mean and sample standard deviation (SD). In the case of very small and large samples, this traditional method is inefficient since it is very complex to select the threshold for identifying the pixels which are affected by noise. This drawback is overcome by using more robust statistics sample adaptive median (AMED) and normalized adaptive median absolute deviation (NAMAD) (Rajaguru et al., 2019). This is defined as:


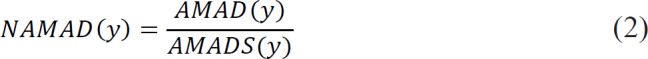


where AMAD denotes the Adaptive Median Absolute Deviation and it can be determined using


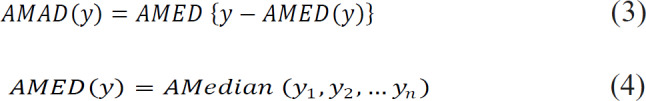


where *AMED* denotes the adaptive median value in the window size of 5 x 5, *AMADS* represents the adaptive median absolute deviation of a standard normal random variable with the value of about 0.6457 and y denotes the vector representation of data. The new statistic mROR is described as


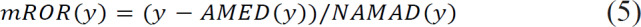


The noise level in each pixel is denoted by mROR statistics. All the pixels in a mammogram image are divided into four clusters according to the mROR value. The most like cluster is formed if the value of mROR is greater than 3, second noisy cluster is formed if the value of mROR lies between 2 and 3, third noisy cluster formation has a condition of value of mROR lies between 2 and 3, and fourth noisy cluster if the value of mROR is less than 1. The lower the value of mROR, noise level of pixel in its neighbours is lower. Thus the cluster with higher value of mROR is selected for further processing. The absolute difference between the processed pixel and adaptive median of its neighbors is computed for the detection of impulse noise; the computed difference is compared with an earlier set threshold in all the four clusters. 

The distribution of pixels based on the value of mROR in mammogram image mdb063 of Mammographic Image Analysis Society (MIAS) database is given in Table 1. It shows the division of four clusters with different noise ratio of 0, 10, 20, 30 and 40 percent. Also few pixels are identified in the most like cluster in the mdb063 image. With the increase in noise density, there is a change in number of pixels in each cluster.

*C. Adaptive Fuzzy Median Filter*

Adaptive fuzzy median filter (AFMF) is developed to overcome the drawback of standard median filter (Hwang and Haddad, 1995). Due to the size of neighborhood is fixed, the performance of standard median filter gets reduced with increase in the variance of spatial noise. But in adaptive fuzzy median filter, the neighborhood size is varied during the process. This adaptation is based on the value of median of pixels in the present window (Ahmed and Das, 2014). The window size is expanded if the value of the median is an impulse. In this paper, the computation of outlyingness of an observation is done according to the adaptive fuzzy median of a sample. If any pixel is portrayed as noisy in any of the two stage, then fuzzy decision based adaptive vector median filtering is made in consistent with the available non-corrupted pixels presented inside the processing window centring the distorted pixel under operation. Thus the adaptive fuzzy median plays a significant role in the impulse noise detection at each level in the mammogram image.

In this paper, detection of impulse noise is first done and then the filtering mechanism is implemented. The detection mechanism of impulse noise is performed in two stages: coarse stage and fine stage. The basic difference between these two stages is the adaptation of threshold to detect the impulse noise. Coarse stage uses relatively larger threshold and fine stage uses smaller threshold for the detection of noisy pixels. Due to the use of adaptive fuzzy median based restored image for subsequent iteration in coarse stage noise detection, the output for next iteration will be closer and closer to the original image after having few iterations. Fine stage uses smaller threshold to identify utmost noisy pixels in an image. Distinct decision rules are then taken on for the detection of noise in four clusters. This detection mechanism is carried out iteratively for better accuracy. Then the filtering process is done by extending the NL-means. This paper uses MATLAB R2017a software tool for the implementation of proposed work.

**Table 1:** Number of Pixels in Four Clusters of mdb063 (512 x 512) with (10-40) % Noise Ratio

Noise Ratio	The most like cluster	Second noisy cluster	Third noisy cluster	Fourth noisy cluster
0%	1915	10,186	48,500	201,543
10%	23,848	7,964	39,087	191,245
20%	47,224	9,242	24,896	180,782
30%	59,753	9,469	21,741	171,181
40%	61,509	12,089	20,112	168,434

**Table 2:** PSNR Values for a Mammogram Image (mdb063) at Different Noise Percentage for Number of CoarseStage Iterations

Iteration #	PSNR at different noise level			
	10%	30%	50%	70%
1	32.6186	31.5761	26.9964	23.8892
2	32.6142	31.5747	26.9971	23.8892
3	32.6115	31.3243	26.9969	23.8996
4	32.6094	31.0547	26.8962	23.8871

**Figure 2:**
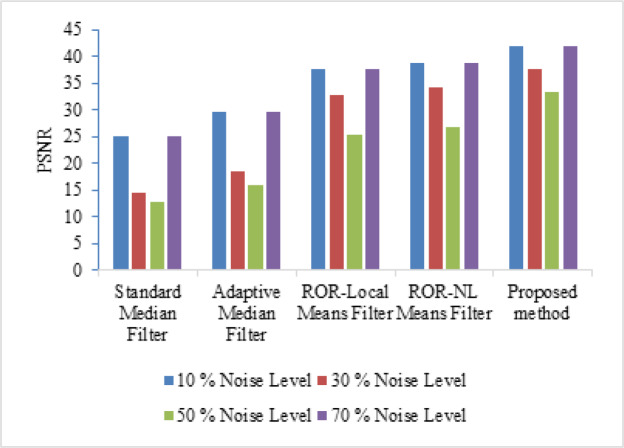
PSNR Comparison of Proposed Method at Different Noise Levels

**Figure 3:**
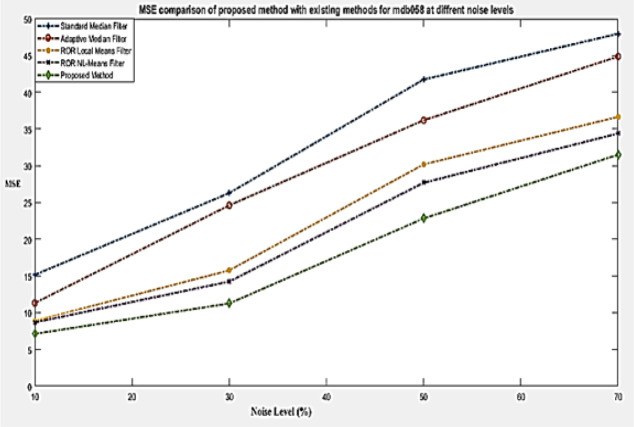
MSE Comparison of Proposed Method at Different Noise Levels

**Table 3:** PSNR Values for a Mammogram Image (mdb063) at Different Noise Percentage for Number of Fine Stage Iterations

Iteration #	PSNR at different noise level			
	10%	30%	50%	70%
1	31.5108	28.3325	24.2131	18.4617
2	30.0142	28.8953	25.2231	18.0878

**Table 4:** Comparison of PSNR of Proposed Method with Existing Methods for mdb058 Mammogram Image

Methods	Percentage of Noise Ratio			
	10	30	50	70
Standard Median Filter	25.14	14.49	12.86	25.14
Adaptive Median Filter	29.53	18.36	16.04	29.53
ROR-Local Means Filter	37.67	32.72	25.39	37.67
ROR-NL Means Filter	38.15	34.19	26.75	38.61
Proposed method	40.68	37.65	33.38	41.91

**Figure 4:**
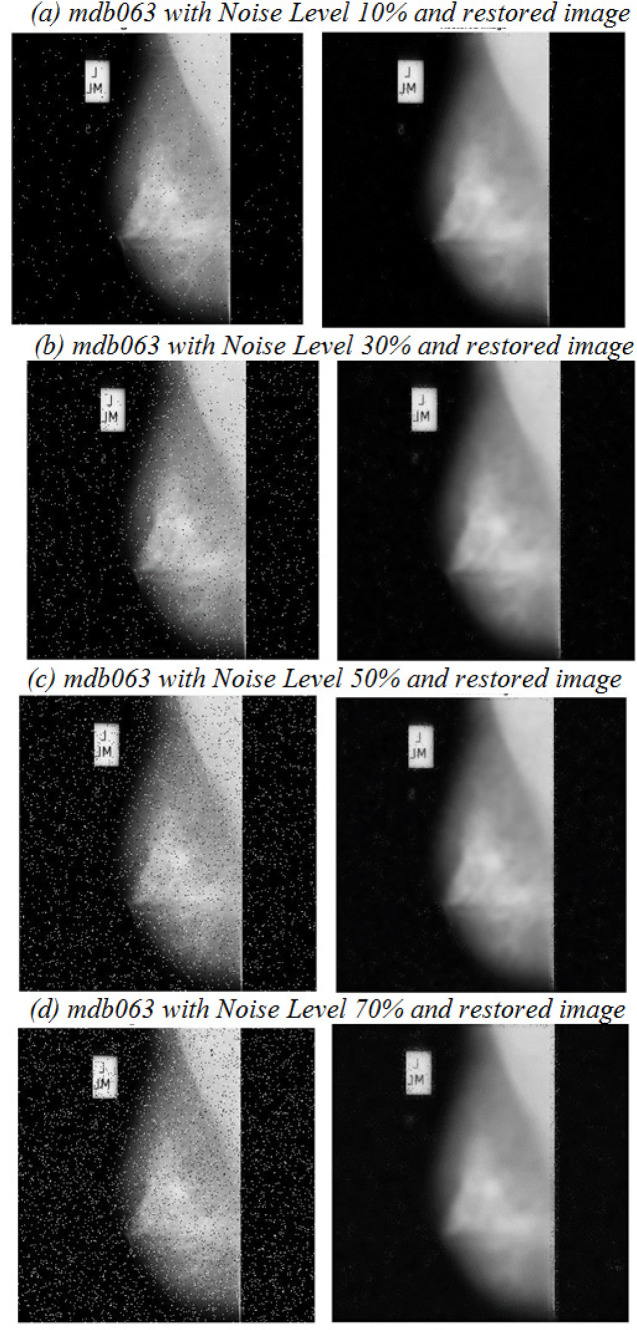
Evaluation of Proposed Method on mdb063 with Different Noise Level

**Table 5:** Comparison of MSE of Proposed Method with Existing Methods for mdb058 Mammogram Image

Method	Percentage of Noise Ratio			
	10	30	50	70
Standard Median Filter	15.16	26.28	41.74	47.96
Adaptive Median Filter	11.32	24.61	36.19	44.87
ROR-Local Means Filter	8.90	15.76	30.18	36.65
ROR-NL Means Filter	8.68	14.24	27.69	34.39
Proposed method	7.12	11.26	22.83	31.51

## Results

The proposed work is evaluated by using mammogram images containing speculated, circumscribed and ill-defined masses in MIAS database (Suckling et al., 1994). The performance of proposed method in terms of PSNR (Peak-Signal-to-Noise-Ratio) and MSE (Mean Square Error) are compared with various existing filters like standard median filter, adaptive median filter, ROR-Local means and ROR-NL means.

Table 2 gives the value of PSNR at different noise level for number of coarse stage iterations of a mammogram image (mdb063). The number of iterations in coarse stage is set as 4 through experimental analysis to have better results as in Table 2. Table 3 gives the value of PSNR at different noise level for number of fine stage iterations of a mammogram image (mdb063). The number of iterations in fine stage is set as 2 through experimental analysis to have better results as in Table 3. If the number of iterations in fine and coarse stage is increased further, then it will resulting in lower PSNR value. Thereby the noise will be dominant over the useful information. Table 4 and 5 give the comparison of PSNR and MSE values of various existing methods with proposed work for mdb058 mammogram image respectively at different noise levels (10%, 30%, 50% and 70%). Correspondingly its graphical representation is given in Figure 2 and 3.

The performance used to assess the mammogram image quality is Peak-Signal-to-Noise-Ratio (Huynh-Thu and Ghanbari, 2008), is defined as:


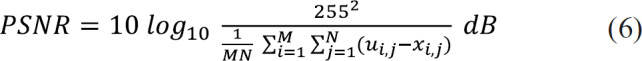


where *u*_i,j_ and *x*_i,j_ represent the value of pixels of the restored and original mammogram images and M, N denotes the size of the image (M×N) respectively. The denominator of equation (6) denotes the estimation of Mean Square Error (MSE) value (Abirami et al., 2016). If the value of MSE is very minimum, then the estimate is closer to the original image. As in Table 4, obtained PSNR value by the proposed method is higher than the existing methods and as in Table 5, MSE value of the proposed method at different noise levels is relatively smaller than the existing methods. For evaluation, the impulse noise ranging from 10 to 70% is taken on the mammogram images. Figure 4 gives an example of a noise affected mammogram image mdb063 (dense-glandular ill-defined mass) with different noise levels and its processed denoised image respectively.

## Discussion

The proposed method implements an efficient denoising algorithm on noisy mammogram images to remove the impulse noise with various density. Modified Robust Outlyingness Ratio (mROR), a new statistic based measure is introduced to compute the outlyingness of pixels and to detect the noisy pixels in a mammogram image, a new detection mechanism has been proposed. Then to denoise the pixels distorted by impulse noise, fuzzy decision based adaptive vector median filtering is extended while not disturbing the noise-free pixels. Extensive simulations indicate that enactment of the proposed work is superior to the current existing filters. Our future work is to modify the proposed algorithm to improve the performance further for real-time clinical mammogram images.
